# Conspiracy theories, science denial, and smallpox. The German-language anti-vaccination movement in the late nineteenth and early twentieth centuries

**DOI:** 10.1017/mdh.2026.10074

**Published:** 2026-10

**Authors:** Martin Tschiggerl

**Affiliations:** Department of Contemporary History, University of Vienna, Vienna, Austria

**Keywords:** Anti-vaccination, Conspiracy theories, German Empire, History of knowledge, Science denial

## Abstract

This article discusses the development of the German-speaking anti-vaccination movement from the second half of the nineteenth century to the early twentieth century as an actor-centred history of knowledge. Although the German anti-vaccination movement was a heterogeneous milieu, recurring shared narratives can be identified within the argumentation of its actors: The movement is characterised by a high affinity to conspiracy theories and an anti-science attitude. Antisemitism and völkish ideologies are also a recurring element of this radical rejection of vaccination in the German-speaking region from the second half of the nineteenth century onwards. These narratives are used to construct a knowledge system of smallpox vaccination that was an alternative to medical science and the political decisions based on it.

## Introduction

On 27 September 1907, the Vienna City Council discussed measures to curb a smallpox epidemic raging in the city, including a potential compulsory vaccination on the model of the German Imperial Vaccination Act of 1874. In a speech on such a motion, the then-mayor of Vienna, Karl Lueger, stated that such compulsory vaccination should be strictly rejected. After all – according to Lueger – everyone knew that far more people die from vaccination than from smallpox itself.^1^ This claim is and was, even according to the knowledge available in Lueger’s time, false, and despite the mayor’s opposition, the smallpox vaccination was widely accepted by the population: according to contemporary sources, up to 20,000 people per day were vaccinated during the pandemic, with vaccines being hastily brought from neighbouring regions and with 500,000 people registered for vaccination.^2^ In his rejection of vaccination, Lueger can be located in a broad anti-vaccination movement in the German-speaking world that has existed since the beginning of the nineteenth century – that is, the beginning of the modern vaccination system. This movement became more and more institutionalised and organised in the course of the nineteenth century and reached its peak at the beginning of the twentieth century.^3^

In this article, I will trace the development of this anti-vaccination movement and analyse its central arguments and positions, as well as look at its ideology and understanding of the world. My key research questions are as follows:

1. What are the central narratives and arguments that have been used within the anti-vaccination movement to dispute the effectiveness of vaccination?
2. How did the opinion leaders of the movement try to establish their own system of knowledge as a counter-discourse to academic–scientific medicine?
3. How strong are the overlaps between the milieus of radical anti-vaccinationists and antisemitic and *völkisch* movements?

The example of Karl Lueger illustrates my main hypothesis: Karl Lueger was not only a radical opponent of vaccination but also a staunch antisemite and spreader of conspiracy theories.^4^ Due to his multifariousness – on the one hand a radical antisemite and on the other a commendable mayor – the person of Karl Lueger is still massively controversial in Austria and the subject of political battles over remembrance. But his opposition to vaccination is largely forgotten. However, it points to an attitude of mind that was typical of the *völkisch* milieu from which he came. I postulate the guiding hypothesis that the German-speaking anti-vaccination movement is characterised by a high affinity to conspiracy theories and an anti-science attitude. Antisemitism is a recurring element of this radical rejection of vaccination, at least in the German-speaking region from the second half of the nineteenth century onwards. While the anti-scientific attitude of the milieu is already well known within research, the repeated recourse to conspiracy theories by anti-vaccinationists already in the nineteenth century has so far been neglected.^5^ The German-speaking anti-vaccination movement is characterised by its deep roots in the *völkisch* milieu and its frequent reliance on antisemitic narratives, setting it apart from similar movements in other countries. This article aims to contribute an innovative perspective to extensive scholarship on historical anti-vaccination movements by situating vaccine opposition within the broader history of disinformation. The article demonstrates that phenomena often perceived as recent developments during the COVID-19 pandemic, such as the intertwining of anti-vaccination attitudes, antisemitic conspiracy theories, and pronounced nationalism, have historical precursors stretching back several centuries. By tracing these historical connections, the article sheds light on the enduring foundations of contemporary anti-vaccination ideologies, thus aligning with the work of scholars such as Christine Binzel and Andreas Link.^6^

The main objective in this article is to investigate the radical rejection of smallpox vaccination. This rejection concerned not only the legal obligation to vaccinate but the health policy technique in its entirety. This distinction is of central importance, especially with regard to the already-mentioned Imperial Vaccination Act of 1874.^7^ Already around the passing of the law, two basic camps of rejection can be discerned in the Reichstag and also in the public debates on the law. On the one hand, there was a rather liberal camp that did not reject vaccination per se but only the legal obligation to vaccinate. These positions are particularly evident in the Reichstag debates themselves: while representatives of the Centre Party tended to argue against vaccination on the basis of individual freedom of choice, Social Democratic politicians tended to refer to class issues and the need for social measures to improve hygiene and public health rather than simply mandatory vaccination.^8^ The second big group of opponents rejected vaccination in its entirety because they considered vaccines useless or even dangerous. So, they went a significant step further in their rejection. This clearly more radical group began to organise itself in various associations on the basis of the Imperial Vaccination Act of 1874, held international congresses, and published a wealth of publications. They are the subject of this article.

Within the German-speaking world, the focus is strongly on people in what is now Germany, as this is where the early seeds of opposition to vaccination were sown. Although opposition can also be found in the Austrian Empire and Switzerland, the movement remained less organised and popular there due to the lack of comparable compulsory vaccination. Vaccination requirements remained only partial in those countries – for example, for school attendance, military service, or in the event of acute pandemic threats. In Switzerland in particular, the population democratically voted against active compulsory vaccination, even though the Federal Council would have liked to enforce it.^9^

## Methodical approach

This analysis is designed as an actor-centred history of knowledge in which I analyse the knowledge system of German-speaking vaccination opponents from the second half of the nineteenth century until the beginning of the First World War, focusing on opinion leaders within the movement. Specifically, the guiding method is a historical discourse analysis following Achim Landwehr in which I analyse different publications of anti-vaccination opponents between 1850 and 1914 to deconstruct a historically specific practice of speaking and thinking about vaccinations.^10^ My main aim is to work out how opponents of vaccination argued in the period I am investigating and how they tried to establish factuality about the topic ‘smallpox vaccination’. The term ‘anti-vaccination movement’ must be understood as a descriptive metaphor and an analytical term, which I use to summarise a markedly heterogeneous milieu. The protagonists of this milieu were recruited primarily from the *Lebensreform* movement and later also from anthroposophy and alternative medical schools (e.g., homeopathy), but also included radical vegetarians, nudists, and animal rights activists.^11^ They were united by the binding bracket of the rejection of smallpox vaccination. Traditional religious reasons, often cited by the general population for not vaccinating their children, played no role among the opinion leaders of the organised anti-vaccination movement.^12^ On the contrary, as I will show in the course of my analysis, they made every effort to present themselves as particularly rational and driven by reason. According to them, it was the pro-vaccination advocates who *believed* in vaccination almost religiously, while they themselves *knew* that it was useless or even dangerous.

From the second half of the nineteenth century, at the latest from the passing of the Imperial Vaccination Law of 1874, they together began to form a counter-public, which grouped itself primarily around a number of different anti-vaccination associations, national and international congresses, and a wealth of publications, such as regular periodicals but also monographs. As a counter-public, the anti-vaccination movement constituted itself primarily through a jointly constructed media space in which its own specific ideas of reality were negotiated. This media space is the source basis for the present study. I analyse statements made within this media space and focus on the central actors of this strongly networked and organised movement. I am therefore less interested in the individual reasons why people have not vaccinated their own children. These were investigated for the German-speaking world in the nineteenth century by Eberhard Wolff, Andreas-Holger Maehle, Philipp Mayr, and Elke Hammer-Luza, among others. These paint an ambivalent picture in which, in addition to purely practical reasons – poor accessibility of vaccination in rural areas, direct costs in the first half of the nineteenth century, and later indirect costs – fear for one’s own child predominated. Rather, my study focuses on those who deliberately sought publicity in order to persuade others not to have their children vaccinated, although I will also consider the interplay between agitation and reception in my analysis. As I will show, the opinion leaders’ arguments are primarily based on ideological reasons stemming from a deep-rooted conflict between different approaches to medicine, health, and the body. However, we should not ignore the fact that there are also monetary interests at play here: Anti-vaccination activists sold books and magazines, charged membership fees for their associations, and admission for their lectures. So, the fight for the (supposed) health of the population was also a money-making venture. This is also evident from a glance at the relevant publications: by the end of the 19th century at the latest, these were also full of advertisements for ‘alternative’ medical practices, spa treatments, and other publications.

The claims within their publications are the source basis of this article. I focus primarily on the publications of five central actors of the anti-vaccination movement as well as on the regularly published journals *Der Impfgegner* (*The Vaccination Opponent*) and *Der Antivaccinator*. These five opinion leaders are: the Stuttgart physician C.G.G. Nittinger, who represented something like a proto-vaccination opponent, the first to campaign monothematically against smallpox vaccination in a total of 25 widely received publications;^13^ Heinrich Oidtmann, who was also a doctor as well as the founder and editor of the journal *Der Impfgegner* and the first key figure of a movement that became increasingly networked from 1874 onwards; the Berlin grammar school teacher, publicist, and politician Paul Förster, who was the founder of the *Deutscher Bund der Impfgegner* (German Federation of Vaccination Opponents), the organiser of numerous international congresses of vaccination opponents, and also played a leading role in the anti-vivisection movement; the grammar school professor, writer, and philosopher Heinrich Molenaar, who was the general secretary of the *Internationalen Impfgegnerbundes* (International Anti-Vaccination League) and editor of the periodical *Der Antivaccinator*; and finally Hugo Wegener, who was an engineer, editor of the journal *Die Impffrage* (The Vaccination Question), and author of numerous books with particularly high circulations, such as *Der Impffriedhof* (The Vaccination Cemetery). In addition to these five main opponents of vaccination, I also analyse anti-vaccination positions in the works of the naturopaths Eduard Bilz and Theodor Hahn, as well as the racial theorist Eugen Dühring.

## Smallpox vaccination and biopolitics

Smallpox, with a historical case fatality rate of about 30 per cent, is considered one of the most dangerous diseases in human history and is responsible for hundreds of millions of deaths.^14^ In the Middle Ages, smallpox became endemic in Europe and significantly determined the development of European civilisations.^15^ The heyday of smallpox began in Europe from the seventeenth century onwards, with around 400,000 deaths annually.^16^ The main victims were children, for whom the disease was particularly fatal. Throughout the seventeenth and eighteenth centuries, at least 10 per cent of child mortality can be attributed to smallpox. Unsurprisingly, the search for protection against the ravages of disease was a recurring motif in many aspects of medical practice.

The earliest attempts to achieve protection are believed to have emerged around the year 1000 in China and subsequently appeared in various regions of Asia and North Africa, in the form of a technique later referred to as inoculation. This procedure entailed introducing material taken from an individual infected with a comparatively mild case of smallpox into the skin of a healthy person through superficial incisions.^17^ People inoculated in this way usually also contracted a mild form of smallpox. However, ‘usually’ means that there was no guarantee of such a mild course – 1–2 per cent of those inoculated had such a severe case of smallpox that they died. Inoculated patients were also contagious after inoculation and could infect other people with smallpox, which could trigger severe epidemic outbreaks of the disease. Nevertheless, the method of inoculation enjoyed some popularity in Europe and the USA in the eighteenth century, starting in England. However, due to the risks and difficulties associated with the technique, its success remained limited.^18^ The big game changer in the search for a definitive protection against smallpox was finally to come from an English country doctor: Edward Jenner.

During his medical work in rural England, Jenner made an astonishing observation: farm workers who already had an infection with the more or less harmless cowpox virus seemed to be immune to the much more dangerous smallpox.^19^ Jenner was not the first to make this observation, but he was the first to systematically analyse and publish his findings. Today we know that an infection with a virus from the orthopox family leads to lasting immunity for all other viruses in this group.^20^ Although viruses and how they functioned were still unknown in Jenner’s time, he drew similar conclusions and undertook an experiment that was decidedly unethical by today’s standards: he infected an eight-year-old child first with cowpox and, after a month, with smallpox. He – and the child – were lucky: there was no outbreak of smallpox, and Jenner’s thesis based on observations was confirmed.^21^ After Jenner published his findings, the technique quickly spread, especially in Europe and North America. In the German states and the Habsburg Empire, too, several doctors began to use vaccination instead of the previous inoculation method.^22^ However, there were some teething problems: since cowpox was extremely rare in some areas, doctors sent each other dried lymph to facilitate the vaccination procedure, and the lymph lost some of its effectiveness in transit. Or, instead of animal material, human material was used by vaccinating from arm to arm, which was associated with risks of infection, since diseases such as syphilis could be transmitted. In the first decades of vaccination, it was also assumed that a single vaccination would guarantee lifelong immunity; only later did it become clear that this immunity would only last for a few years and that it was therefore necessary to vaccinate at least twice in a lifetime.

Despite these initial difficulties, several German states decided to make vaccination compulsory: the Grand Duchy of Hesse and Bavaria in 1807, Baden and the Electorate of Hesse in 1815, Württemberg in 1818, and Hanover in 1821.^23^ In Prussia, a regulation was in force from 1835 that provided for compulsory smallpox vaccination in the case of acute pandemic threats; compulsory vaccination was carried out in the military from the beginning of the century, and from the 1830s on, re-vaccination was also compulsory.^24^ After the unification of the German states, the Imperial Vaccination Act of 1874 adopted an overall solution for the whole of the German Empire in the form of compulsory smallpox vaccination for children up to the age of one and a revaccination at the age of twelve.^25^

This legally prescribed state intervention in the individual bodies of its citizens in favour of collective health is situated in the conglomerate of health, medicine, power, control, and knowledge that Michel Foucault referred to as ‘biopolitics’ or ‘biopower’.^26^ The German Empire in particular proved to be the ‘experimental field of modernity,’ where vaccinations were rolled out widely at a very early stage.^27^ Foucault located vaccinations at Europe’s transition to modernity and even dedicated a separate seminar to it to accompany his lectures on ‘biopower’. They stand at the end of a ‘trail of infection’ that can be traced through Foucault’s work, following Philipp Sarasin, along which the ways in which the state has reacted to epidemic or pandemic events or – and this is the crucial point in the case of vaccinations – attempts to prevent them in advance are grouped.^28^ Foucault used political reactions to pandemic threats to illustrate the genesis of modern state power and divides this development ideally into three sequential steps. At the end of these developmental steps is a model, which Foucault illustrated using the example of smallpox and the smallpox vaccination. It is a system of ‘security dispositives’ that has given up the disciplinary dream of total surveillance of the population but still aims to contain pandemics and epidemics through ‘medical campaigns’ – such as vaccinations or the collection and statistical analysis of data.^29^

From the second half of the eighteenth century onwards, a process can be observed that is outlined in research as medicalisation.^30^ This concept encapsulates social developments in which academic medicine succeeded in attaining a hegemonic position through a close connection with state power and in ‘imposing’ its medical services on broad sections of the population. This process of ‘medicalisation’ was initially described as a top-down movement – that is, a powerless population became the object of a top-down subjugation to biopolitical techniques and control instances. Even though this interpretation cannot be dismissed out of hand in many respects, more recent research presents a somewhat more differentiated picture and also acknowledges the agency of citizens, who were already able to act as consumers of medical services in the eighteenth century.^31^ As already mentioned, the struggle for discursive hegemony and especially the role of the population as objects of biopolitical techniques to control public health are central aspects within the framework of ‘vaccination opposition’.^32^ The attempt to control and ideally contain infections within the population must be understood as part of the recognition of disease as a ‘political problem’ that calls for a political solution.^33^ As Francisca Loetz has shown, the concept of ‘medicalisation’ and thus also the focus of research on this process differs greatly between Germany, France, and England. The German perspective is strongly influenced by Ute Frevert, which has led to a strong focus on the political power component of the absolutist state and its bourgeois public sphere, as well as the practices of resistance against it.^34^ This focus can also be seen in the debate on the history of German-speaking anti-vaccination movements to date.

After all, this state action did not take place without a reaction from at least part of the population. The fear that state authorities, private institutions, or other powers exercise too much control over people’s individual lives and bodies is a recurring metanarrative in the rejection of biopolitical measures and techniques; many countries were confronted with this very phenomenon in the face of the COVID-19 pandemic.^35^ Especially where the previously voluntary vaccination against smallpox became compulsory, a particularly strong resistance to it formed. Therefore, the Imperial Vaccination Act of 1874 acted as a kind of catalyst for the publication of anti-vaccination materials and the increasing institutionalisation of opponents in organised associations and in the form of regular networking at congresses. In the following, I will show the central arguments within this knowledge system.

## Deadly risks for your child and your nation

In their various publications, the most frequent and popular argument of the vaccination opponents studied was a strong (over)emphasis on the risks and dangers of vaccination. These would clearly outweigh the benefits, which, depending on the position, would be scant or even non-existent. C.G.G Nittinger created the blueprint for this central strand of discourse on radical rejection of vaccination: in his opinion, vaccination was the same *Gift* (toxin) as smallpox itself.^36^ According to Nittinger, vaccination triggered the disease in the first place, and a particularly severe form at that – vaccination itself was therefore the reason for the decline of the German people. Through the wanton poisoning of vaccination, the military strength, the ability to procreate, the intelligence, even the appearance of the people would be damaged.^37^ Here we already encounter the portrayal of smallpox vaccination as a national threat that harms the people as a whole. In this argument, Nittinger is essentially a proto-*völkish* anti-vaccinationist. A national ideology was therefore practically ingrained in German anti-vaccination sentiment from the outset. The common assessment of Nittinger in previous research^38^ as a ‘scurrilous’ outsider on the fringes of the movement must be put into perspective insofar as a systematic analysis of his publications shows that he anticipated many of the arguments of later opponents of vaccination. Nittinger created something like an outline for the radical rejection of vaccination and was widely received and quoted within the movement after his death in 1874.^39^ Especially among those who saw vaccinations as harmful not only to individuals, but to the entire nation.

Paul Förster in particular argued quite similarly, but he developed Nittinger’s picture of the poisoning of the people, locating it even more strongly in a *völkisch* ideology. According to Förster, vaccination is a ‘corruption of the nation’s body’ and a ‘poisoning of the whole people,’ which has been forced upon them by a small minority.^40^ In addition to the poison, he focused even more than Nittinger on the aspect of blood and the idea of a pure, German blood that would be poisoned by vaccination. Here we encounter the connection with blood and soil, essential in *völkisch* ideology, prominently in the negotiating arena of the anti-vaccination movement. Förster emphasised that the ‘preservation and regeneration of pure blood’ is the most urgent task of medicine, but that the idea of vaccination runs counter to this.^41^ That this ‘German’ blood had to be kept pure and protected from foreign influences was perhaps Förster’s most central argument in practically all his published texts, even those not directly related to vaccination. But even in what is perhaps his most popular text, *Deutsche Bildung, deutscher Glaube, deutsche Erziehung*, an educational manifesto deeply rooted in *völkisch* ideology, Förster ends his appeal to the German people to act in the right way with a plea to end the ‘superstition of “protective” vaccination’.^42^ Förster can be seen as an almost archetypal example of the most extreme wing of the *völkisch* part of the anti-vaccination movement. He explained his rejection of smallpox vaccination based on the necessity to protect German blood from an outside influence that would damage it. This combination of *völkisch* ideology, alternative medical theories, racism, and (as I will show later in this article) antisemitic narratives was not an isolated case within the *Lebensreform* movement, to which Förster clearly belongs.

For both Nittinger and Förster, the danger of vaccination was a rather abstract one; it threatens the totality of an imagined, cohesive *Volkskörper*. The individual risk of vaccine harm as an actual risk of vaccination played a rather subordinate role for both authors. Rather, they used it as a conduit for the danger to the entire nation. In contrast, the works of Oidtmann, Molenaar, and Wegener may have been much more in tune with the lives of contemporary parents, who may have been less interested in the blood of the German people per se than in the safety and well-being of their own children. This is also demonstrated by Eberhard Wolff in his study of vaccine refusal in Württemberg, and by Elke Hammer-Luza in her study of Styria in Austria. Fear for the health of one’s own children is the dominant reason, while ideological factors play a subordinate role.^43^ The extent to which concerns about risks were influential is evident in government vaccination campaigns, which were precisely aimed at combating this fear. Anti-vaccination arguments also exploit this fear. Oidtmann even gave one of his first publications the subtitle ‘Why I don’t vaccinate my children’ and listed the risks of vaccination as the primary reasons: he wanted to save his children from all the harm the vaccination would bring. For his beliefs and above all for the protection of his children, he was even prepared to be charged and tried. In addition, he underpinned the individual risks with figures and data on the risks to society as a whole. The fear of vaccination clearly outweighed the fear of the disease.^44^ Wegener emphasised this fear particularly strongly in his publication *Der Impffriedhof*.^45^ In it, Wegener listed a total of 36,000 (alleged) vaccination injuries, mainly from German-speaking countries but also from England and the USA, and illustrated them with 139 pictures. The damages range from fever and rash to death. The authenticity of the individual cases or of various details within them can no longer be verified today. What is certain is that vaccinations in the late nineteenth and early twentieth centuries were associated with a significantly higher risk than in the present. Poor hygiene or contaminated vaccine material as well as the sometimes-unfavourable overall health situation of vaccinated persons led to a fundamentally significantly higher probability of suffering vaccine damage than today.^46^ Criticism of individual aspects of vaccination, particularly by those opposed to vaccination, has led to an improvement in hygiene and safety standards. In the German Empire, for example, from the 1880s onwards, due to the risk of syphilis transmission, vaccinating from arm to arm was abandoned, and only material from cows was used. This also represents one of the few successes of the German anti-vaccination movement – while the British movement, for example, succeeded in obtaining an exemption from compulsory vaccination through a conscience clause, German anti-vaccinationists were denied such a concession, at least in the nineteenth century.^47^

Overall, it can thus be concluded that vaccination opponents massively overstated the risk of vaccinations. To this end, they used individual cases to portray vaccination in its entirety as dangerous and, above all, more dangerous than smallpox itself. The most prominent of these cases, at least at the beginning of the twentieth century, was that of the child Willy Otto from Elberfeld, who was mentioned in several publications opposing vaccination.^48^ According to these publications, Otto was vaccinated on 17 May 1909, fell ill with a feverish rash on 25 May, and died of encephalitis on 6 June. Whether the smallpox vaccination was causally responsible for the child’s death can no longer be determined today – it may have been, as encephalitis was a possible, albeit very rare, side effect of smallpox vaccination. More important is how Otto’s death was presented in the relevant publications: the authors positioned the case particularly prominently in support of the core argument of their text and also provided a picture of the child on his deathbed, the cuts of the smallpox vaccination clearly visible. As Christine Arndt has already shown, opponents of vaccination combined the possibilities of mass media dissemination with the use of images in their publications to stir up fear and thus achieve a broad rejection of vaccination.^49^ In doing so, they were able to dock onto existing insecurities in the population and create factuality precisely through the use of the (supposed) evidential power of images: the tragic individual case – regardless of whether it was real – became a conclusive argument against vaccination per se through the combination of text and image. Children, as a group particularly worthy of protection, were especially affected by compulsory vaccination and the potential dangers of vaccination, regardless of whether these risks were real or imagined. Therefore, anti-vaccination propaganda was also aimed precisely at the supposed protection of children. The child as a battleground around vaccinations functions on two different levels. First, on a practical level: concern for one’s own child is probably the central motivating factor in parents’ vaccination decisions. Jens Oliver Krüger and Christine Freytag, for example, have already demonstrated this using the example of educational guides from the eighteenth and nineteenth centuries on variolation and later vaccinations: Is it worth exposing your child to risk through a conscious action (vaccination), or is it better to follow the natural course of events and not interfere with this supposed order yourself? Even though vaccination carried a significantly lower risk than variolation, the debates surrounding it remained very similar.^50^ On a second, symbolic level, however, children also represent the future, hope, and collective progress itself, and the meaning of politics must be aligned with their ‘protection’. The child as a sacred figure is used to generate morality – moral politics must be measured by its well-being.^51^ The slogan ‘Save Our Children’ as used, for example, in the 1970s by a homophobic counter-movement against the fight for gay rights, thus serves as a symbolic code that acts as a pretext for maintaining the normativity of a supposedly natural and moral order.

In this two-layered approach, vaccination opponents were helped by the paradox that the number of people infected with smallpox fell due to rising vaccination rates – smallpox lost its horror as a great child killer, and vaccination could be staged as a new spectre of terror. We encounter this phenomenon also in recent anti-vaccination campaigns: infectious diseases (e.g., measles) become rarer due to the availability of vaccinations, the willingness to vaccinate decreases due to the lack of awareness of the danger of the disease, and the disease returns.

## Statistics, contagion, and science denial

How and why vaccinations worked was not clearly verifiable and provable for contemporaries. For much of the nineteenth century, the theory of contagion was only one medical explanatory model among many; Pasteur and Koch did not postulate their germ theory until the last third of the century, and the smallpox virus itself could not be detected until the beginning of the twentieth century.^52^ Who can claim factuality within the discourses of the time and in what way within the discussion surrounding smallpox vaccination is therefore a decidedly important question. What constitutes good science, how it works, and what its results are is a major battleground of debates between vaccination proponents and opponents. The anti-vaccination movement can be seen as part of a milieu critical of scientific medicine, as described by Martin Dinges for the late nineteenth century.^53^ The critic is characterised above all by struggles for sovereignty between non-professionals and scientific medicine, but also between doctors who still adhered to traditional healing and diagnostic methods and those with an academic-scientific approach.^54^ This is also shown by a look at the protagonists of the anti-vaccination movements we analyse: complete non-specialists like Förster, Molenaar or Wegener, doctors like Nittinger and Oidtmann and ‘alternative’ practitioners like Hahn or Bilz. In the last third of the nineteenth century in particular, there are clear signs of the triumph of academic medicine, at least in terms of power politics, and from this point onwards it had a monopoly on medical explanations. The passing of the Imperial Vaccination Act of 1874 in particular is a symptom of this success, and the organised resistance to vaccination can therefore also be interpreted as a rearguard action, possibly also triggered by the fear of a perceived loss of significance in the public struggle for biopolitical power. At the same time, this law further fuelled the professionalisation and scientific monitoring of medicine. After all, the observation of vaccination, the establishment of medical statistics and, as part of it, vaccination statistics, was one of the arguments that led to the founding of the Imperial Health Office and was one of its core tasks.^55^

However, it would be too simplistic to accuse the opponents of vaccination of being fundamentally hostile to science as a whole, but to scientific medicine. For example, as early as 1901, the medical historian Paul Kübler, in his work on the history of smallpox, stated that the German anti-vaccination movement was ‘decidedly hostile to scientific medicine’^56^ or, as the physician Martin Kirchner puts it somewhat more benevolently, at least ‘outside of scientific medicine’,^57^ a judgement shared by later historical works on this movement. Kirchner himself clearly stood on the other side of the divide; after all, he was Privy Council (‘Geheimer Obermedizinalrat’) in the Prussian Ministry of Interior and one of the leading medical officials in Prussia and the German Empire.

The battle for factuality was carried out in the scientific language of the time. Porter argues that statistics emerged in the nineteenth century as an attempt to objectify the world in the form of mathematical data and thereby make it more understandable and calculable.^58^ The German government and academic medicine used this ‘technology of trust’^59^ to prove that vaccinations were overall useful and mostly harmless.^60^ However, opponents of vaccination did the same – sometimes even with the same statistics – to prove that vaccinations were useless and even dangerous. This shows a completely different interpretation of data based on an opposite intention. Here, too, Nittinger provided the prototype of the argumentation by trying to prove with the help of statistical data that vaccination was not only useless but would even spread smallpox in the first place.^61^ It is not important for this study whether he was right in his argumentation, but it is remarkable that he used the same techniques used by the proponents of vaccination. In this way, Nittinger’s publications give the impression of scientificity: they are full of tables, graphs, and rows of figures in which child and infant mortality, smallpox, typhoid, and syphilis cases, and infection and death figures are supposed to paint an overall picture that clearly proves the harmfulness of vaccinations. Here we encounter a technique that is referred to as cherry picking in the description of science-sceptical arguments in the twentieth century: statistics and case studies are arbitrarily selected to prove one’s own position. Counterevidence is ignored or labelled as false and a lie.^62^ Part of this is the emphasis on anecdotal individual cases as evidence, such as that of Willy Otto from Elberfeld, which is used to illustrate the horrors of vaccination.

This strategy of using numbers and data to establish factuality about the damage caused by vaccination runs through the entire nineteenth century. It is particularly evident in the journal *Der Impfgegner*, edited by Oidtmann.^63^ Practically every issue of the journal, which appeared with varying frequency between 1876 and 1914, contains more than one article on statistics. There may also have been a certain target group relevance here: as Andreas-Holger Maehl has shown, the vast majority of the magazine’s subscribers had at least an academic education. Scientific-sounding arguments seem likely to have been particularly successful with them.^64^ Oidtmann tried to claim interpretative sovereignty over the numbers: official figures were doubted, the advocates of vaccination accused of misinterpreting or even faking them to cover up the risks. It is important to note that the overall statistical situation regarding the pros and cons of vaccination was very desperate, even for the last third of the nineteenth century, as there was no standardised record of smallpox cases, vaccination coverage rates, and vaccine damage.^65^ Neither the advocates nor the opponents of smallpox vaccination were therefore able to come up with any actually reliable data from today’s perspective. However, the government’s arguments in favour of the success of vaccination were clearly more robust than the counterarguments of vaccination opponents. Even from today’s perspective, the most problematic aspect of the statistics was not the fact that the figures did not show a decline in smallpox after the introduction of vaccination, but rather that vaccination damage could not be systematically recorded. In the vacuum of definitive scientific explanation of how and why vaccination actually works, there was much room for different interpretations of an inherently thin database. Both camps apparently agreed that data should form the basis for decision-making. Opponents of vaccination may have attempted to adopt and replicate the arguments used by proponents of vaccination. After all, since the early nineteenth century, infection and mortality rates had been the dominant justification for vaccination. The fundamental problem for vaccination opponents was that they were arguing from an outsider position – vaccination was an established practice, and from the second half of the nineteenth century onwards, academic medicine began to assert itself as the orthodox model of knowledge with its teachings on infection and germ theory.

That this battle for interpretive sovereignty was not conducted with kid gloves can be seen in the terms the adversaries used to refer to each other. The opponents of vaccination very often used the terms ‘superstition’ and ‘foolery’ to describe vaccination in particular and academic–scientific medicine in general. This is a clear devaluation that locates the opposing side in pre-modern magical thinking, and it is precisely the same accusation with which the opponents of vaccination have repeatedly found themselves confronted. The accusation of ‘hostility to science’ thus applies to these opponents of vaccination insofar as it was not scientific methods that were fundamentally rejected but the positions of hegemonic medical science. Förster even goes so far as to argue that academic medicine is not a science at all.^66^ In the tradition of the life reformers, he uses the pejorative term *Schulmedizin* (school medicine/orthodox medicine) to classify his opponents, a term that is still used today within alternative medical currents in German-speaking countries.^67^ The core of the argument is a fundamental rejection of the theory of infection and the idea that diseases can be cured or even prevented in advance by administering medication. Both are essential components of the academic–scientific medicine that was just becoming established in the last third of the nineteenth century. The main disagreements between the proponents and opponents of ‘modern’ medicine lie in the different understandings of how the human body works, how diseases develop, and how they can best be treated. As the radical opponents of vaccination in particular recruited very strongly from the *Lebensreform* movement and associated ‘alternative’ medical movements, their ideas of bodies, illness, and healing were also very strongly influenced by these positions. Even though the *Lebensreform* movement must be understood as a very heterogeneous and constantly changing group, the common thread can be described as a social reform initiative aimed at promoting a healthier, more natural, and self-determined lifestyle. ‘Nature’ was defined primarily as a contrast to urbanisation, industrialisation, and modernity, in the sense of a natural state of humanity that must be regained. The overarching goal was to achieve a state of harmony between physical and mental health, which was considered the ideal for individuals and society as a whole. The movement sought to combat (perceived) social issues by advocating a return to nature and simplicity, overcoming industrialisation and mass consumption. While the movement was not ideologically uniform, some strands were influenced by eugenics and racist theories emphasising the concept of a homogeneous national or ethnic community bound together by traditional values such as honour, virtue and duty.^68^ Particularly with regard to classic antisemitic narratives, it is hardly surprising that the decaying city, dirty industry, and alienating modernity were often associated with the Jews. Vaccinations, as a symbol of modern medicine that decisively interferes with the natural order, were a focal point of rejection – after all, it was not vaccinations that offered protection against disease, but rather the right, natural lifestyle. That, at least, was the thinking of anti-vaccinationists and Lebensreformers.

The founder of the magazine *Der Impfgegner*, Theodor Hahn, was one of the most important figures in the early naturopathic and alternative medicine movement in German-speaking countries.^69^ Hahn united in his person many of the schools of thought from which a good part of the organised anti-vaccination movement was recruited in the late nineteenth century: he was a natural healer with a particularly strong focus on water cures and gymnastics, a convinced vegetarian and, as an animal rights activist, also a declared opponent of vivisection, and even active in the labour movement. His opposition to vaccination was probably based on a deep conviction and many years of practicing alternative medicine. In the *Impfgegner* itself, there are advertisements for alternative medicine doctors, associations, and publications in practically every issue. This cooperation took place in both directions. Friedrich Eduard Bilz, for example, whose *Das neue Naturheilverfahren* sold a total of 3.5 million copies and who is considered one of the most influential naturopaths of the late nineteenth century, postulates in his bestseller that vaccination is useless and dangerous, and in it he advertises the *Deutscher Bund der Impfgegner*.^70^ Bilz is only one example of the strong overlaps between radical vaccination opponents and naturopaths: opponents of vaccination assumed an inherently healthy and natural body that was made ill in the first place by vaccination.^71^

In this perspective, protection against smallpox was not offered by vaccination but by the right way of life – just as the *Lebensreform* movement proposed.^72^ If a person becomes ill in spite of this, treatment should take place according to the guidelines of naturopathy, not according to the guidelines of academic medicine. Common to this position is an absolutism of nature and a supposedly natural life that is placed in contradiction to modern, scientific life. Humans cannot and, above all, must not intervene in this natural life. Parts of the anti-vaccination movement assume an ensouled nature, in which nothing happens by chance, but everything has its meaning and purpose – even children dying of smallpox. Here we encounter the anti-modern attitude of the radical anti-vaccination movement: man must not live in opposition to nature. Modern life – urbanisation, technification, scientification, industrialisation – only distance humans from nature. Vaccinations are perceived and rejected as the epitome of this modern life.

The social Darwinist approach in this thinking, which sees illnesses and also dying from them on the one hand as natural and inevitable and on the other hand as the weakness of those affected, is not difficult to recognise, especially when looking again at Förster’s work. Even stronger than Oidtmann before him or Molenaar and Wegener after him, he also argues in terms of survival of the fittest: modern medicine would enable the weak, who would have died in nature, to survive. Here, a particular inner contradiction can be seen within Förster’s argumentation: on the one hand, vaccination is useless, even dangerous; on the other hand, it interferes with natural selection, which in fact presupposes its effectiveness.^73^

In the process, the actors studied develop their own explanatory models for the occurrence and also the supposed disappearance of smallpox. Oidtmann, for example, posits the thesis that smallpox was caused by contaminated sheep’s wool – especially sheep’s wool that came from outside Europe. He explains the decline in smallpox after the introduction of vaccination by the decrease in the use of sheep’s wool and the improved disinfection of this wool. According to him, vaccination had nothing to do with this decline in smallpox cases. On the contrary, he says, academic medical science, and with it the entire doctrine of contagion, is a dangerous and wrong path.^74^ Oidtmann’s claim can be located in the medical miasma theory. According to this theory, diseases were caused by bad smells and a lack of hygiene – the dirt in sheep’s wool and the lack of cleanliness of the so-called *Lumpen* (rags) had caused smallpox. He thus clearly adheres to a pre-modern idea of disease, which was, however, still widespread in the nineteenth century and was only slowly replaced by the theory of contagion and germ theory.^75^ Nittinger had already used this explanatory model before him. However, he did not believe that wool was responsible for the decline in smallpox, but rather the weather: he was convinced that wet and cold weather caused the disease, while warm and dry weather prevented it. He therefore explained the falling infection rates with the rise in temperature. Instead of infection theory, meteorology served as a point of reference.^76^ In general, German anti-vaccinationists of the nineteenth century were characterised by their opposition to first the contagion theory and then the germ theory; in their medical explanations, they resorted to theories that were in decline at the time, such as the miasma theory.

In their rejection of academic–scientific medicine and to delegitimise its explanatory models, the vaccination opponents studied repeatedly resorted to the argumentative use of conspiracy theories.

## Conspiracy theories and antisemitic narratives

Julia Nebe et al. postulate in their knowledge–historical analysis of Förster’s work that the German-language anti-vaccination movement differs from comparable movements in other countries primarily in its repeated recourse to antisemitic conspiracy theories.^77^ Indeed, antisemitic conspiracy theories are a recurring motif within numerous anti-vaccine publications during the period studied. Quite early, Nittinger resorted to blaming the Jews for the spread of vaccinations. In this context, a footnote on page 37 of his first monograph is striking: in the continuous text, Nittinger states in reference to the ‘medical aristocracy’ that the ‘exaggerated fear of this institute’ had made the spread of smallpox vaccination possible, since people had gotten vaccinated out of fear. In a footnote after ‘institute,’ he adds, ‘שקר עד of the Jews,’ which can be translated as ‘false testimony’ or ‘lying’.^78^ This suggests – albeit covertly and cloaked by the use of Hebrew – a Jewish conspiracy through which the medical technique of vaccination could become so widespread in the first place. It should be noted here that even for the time when the texts were written, Nittinger is conspicuous for his decidedly cloistered language. He regularly resorts to foreign-language interpolations and a writing style strongly dominated by metaphors. It can therefore be assumed that his writings were primarily aimed at an educated audience.

**Figure 1.**
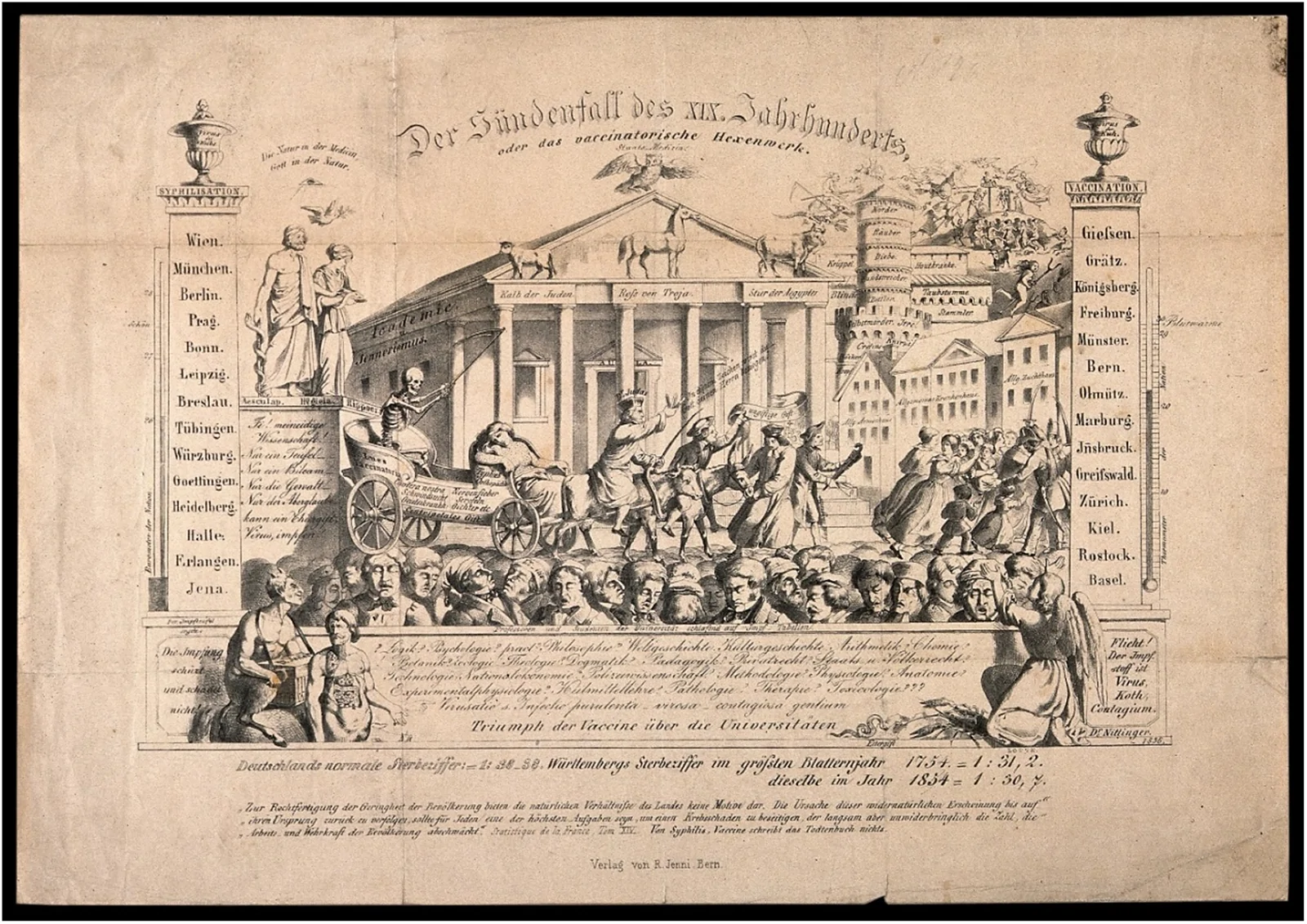
Lithograph after C.G.G. Nittinger, 1856. Figure 1. long description.

This antisemitic implication is not an isolated case in Nittinger’s argumentation. Beginning in 1856, for example, he circulated a painting entitled ‘The Fall of the 19th Century or the vaccination witchcraft,’ which uses a motif from the French anti-vaccination movement and shows a procession of vaccination advocates leading a wagon carrying a woman, obviously sick with smallpox, and Death past a university. However, Nittinger made an important change to the motif in the centre of the painting, which distinguishes it from the French original: the driver of the carriage has ‘Dr. Judas’ written above his head, and in the listing of various idols on the temple-like building of the university, the ‘Calf of the Jews’ is positioned on the far left (Fig. 1).

Nittinger’s antisemitism did not exist in a vacuum, but must be understood as part of the strong overlap between *völkisch* and anti-vaccine positions mentioned earlier. The extent of these overlaps is shown by a line in Eugen Dühring’s proto-text of a modern, racially legitimised antisemitism, *Die Judenfrage* (The Jewish Question), from 1881. In this line, which received relatively little attention in research until the COVID-19 pandemic, he states that everyone knows that vaccination is a plot by Jewish doctors to make money, supported by Jewish politicians and publicists.^79^ As Nittinger’s example illustrates, the association between radical anti-vaccination discourse and antisemitic conspiracy narratives constituted a recurring motif within the German anti-vaccination movement of the nineteenth and early twentieth centuries. As recent studies on the organised opponents of COVID-19 mitigation measures – who self-identified as Querdenker (‘lateral thinkers’) – demonstrate, this connection has also resurfaced in the twenty-first century.^80^ This becomes even clearer than in the case of Dühring – whose anti-vaccination is only a small side note in a thematically different main work – in the person of Förster. He was not just the founder of the German Federation of Vaccination Opponents and one of its most important spokesmen but also a signatory of the antisemitic petition of 1880/81.^81^ Förster’s anti-vaccination agitation is strongly rooted in his antisemitic, *völkisch* ideology. This was based on the idea that the German race, and in particular German blood, must be protected from Jewish influence and the poison of vaccination. The association between Jews and poison draws on narratives that have been handed down for centuries in Europe and especially in German-speaking countries, for example, in the form of the medieval narrative that Jews poisoned the wells and thus triggered the plague, which caused pogroms against the Jewish population in various places in the German-speaking world.^82^ The accusation of targeted infanticide can also be located in traditional, antisemitic tales. The fear of a negative Jewish influence was a determining factor in the work of Förster as well as the *völkisch* movement. Vaccinations were seen as one such Jewish plot and at the same time rejected as an interference in natural life.

This combination of anti-vaccination, naturopathy, *völkisch* ideology, and antisemitism remained a dominant phenomenon in German-speaking countries even after the First World War. It experienced a strong upswing with the rise of the National Socialists in the Weimar Republic. Leading protagonists of the Nazi state, such as Rudolf Hess, Julius Streicher, and Heinrich Himmler, were convinced vaccination opponents and supporters of naturopathic and alternative medical ideas.^83^ With the *Neue Deutsche Heilkunde*, there was even an attempt to combine homeopathy and other alternative medical techniques with scientific–academic medicine, but this effort failed.^84^ The movement, explicitly promoted by Rudolf Hess as an alternative to the so-called ‘Jewish-Marxist’ academic medicine, sought to merge scientific methods with naturopathic and alternative medical practices in response to a perceived ‘crisis of medicine’. Advocates of the *Neue Deutsche Heilkunde* criticised the mechanisation and medicalisation of modern science, framed vaccination as a dangerous product of the alliance between state and medicine, and called for a holistic, nature-based approach as the solution to this crisis. Despite the anti-vaccination stance of some Nazi officials, the Nazi state itself also relied on a broad-based vaccination campaign and a high vaccination coverage rate among the population.

Besides obvious antisemitic conspiracy narratives, the radical rejection of vaccination, at least in the period studied, has a fundamental affinity with conspiracy theories. The vaccine opponents used conspiracy theories to explain how it was possible for vaccinations to spread at all, despite their uselessness or danger, and how the deaths and illnesses caused by vaccination were kept secret. Conspiracy theories can be defined as narratives that explain historical or contemporary phenomena as the result of a conspiratorial plan by a particular group or individuals.^85^ They are cohesive, mono-causal, and self-referential theories that regard all explanatory models lying outside their own narrative as part of the very conspiracy they seek to uncover. In the logic of conspiracy theories, nothing happens by chance, but they create connections and causalities where none would be found without them.^86^ According to Cubitt, conspiracy theories assume an intentional conspiracy to achieve a specific plan, are characterised by a dualistic worldview that clearly divides the world into good and evil, and make an occultist distinction between a visible world and a secret one that needs to be uncovered.^87^ All of the vaccination opponents studied use such theories to imply a secret plan of academic medicine; the only difference among them concerns the imagined intention of academic medicine. Whilst Nittinger and Förster suggest a secret plan to poison the people, others ‘merely’ suggest a secret plan to conceal the dangers of vaccination.

Oidtmann, for example, in his opening speech at the International Anti-Vaccination Congress at Charleroi 1885, which was published nearly 30 years later by Molenaar, spoke of a ‘vaccine-ritual mass murder’ that had been systematically concealed in the medical reports. He relates this to ‘Jewish ritual infanticide’ and the outcry that this – as he writes unfoundedly – would have caused in international media, whereas the mass infanticide through vaccination seems not to interest anyone.^88^ However, he does not see the intention of the vaccinators as planned murder, as Förster does, but as a business model that is designed to make profits while concealing the immense risks of vaccination. To this end, doctors would, among other things, falsify the death certificates of children who had died from the vaccination. This kind of support makes it clear that the official statements about smallpox vaccination are not to be trusted and that it is in fact much more dangerous than postulated. This combination of over-emphasising the risks of vaccination while insinuating a conspiracy to cover it up is probably the most recurring narrative within the publications studied. Through this othering of the negatively connoted orthodox medicine, a separate group of ‘insiders’ is also constructed. In other words, those who know about the ‘true’ nature of vaccinations must look behind the scenes of falsified health data and share their knowledge in publications. However, a certain economic interest can also be assumed. Similar to conspiracy theorists in the present, the anti-vaccinationists of the late nineteenth and early twentieth centuries also profited from the sale of their books and journals as well as their lecturing activities.

Another example by Theodor Hahn shows how strongly the recourse to conspiracy theories was used as a central argument in the attempt to convince and gain attention. Under the pseudonym ‘Dr. med. H. Hennemann’, he has written additional anti-vaccination publications, some of which are much more radical in tone than his other works. Particularly important in relation to conspiracy theories is the book *Die Schlimmsten Jesuiten des deutschen Reiches und des deutschen Reichstages*, which, according to its subtitle, is addressed directly to Otto von Bismarck in his function as Chancellor of the Reich.^89^ Here, Hahn tries to jump on the anti-Catholic bandwagon of the German *Kulturkampf* and explain why academic medicine is basically the same as the particularly strongly antagonised Jesuits, as both would advocate a secret plan to destroy Germany. While the Catholic Church is only responsible for the deaths of hundreds of thousands of people, scientific medicine has millions, if not billions, on its conscience, according to Hahn.

## Conclusion

Although the German-speaking vaccination opponents in the long nineteenth century were a heterogeneous milieu, recurring shared narratives can be identified within the argumentation of their actors. In my analysis of their knowledge system, I was initially able to identify and describe in more detail two strands of discourse that have already been documented in previous research:

1. The overemphasis of the risks of vaccination, which was portrayed as a threat to the individual bodies of the children to be vaccinated on the one hand, and as a collective danger to the people as a whole on the other.
2. The attempt to establish their own knowledge system as a counter-narrative to scientific medicine. This was closely linked to life reformist theories and practices with strong leanings towards the völkisch milieu.

However, I was also able to show that these two strands are highly dependent on a third factor that has been neglected in research until now:

1. The recourse to conspiracy theories, which were partly combined with antisemitic narratives.

These three strands are closely interwoven to construct a reality of smallpox vaccination that is an alternative to medical science and the political decisions based on it. But the first two strands would not work without the third one: The vaccination opponents claim that vaccination is dangerous, contradicts the way the human body really works, and is part of a great conspiracy of academic–scientific medicine. Even within the radical wing of the German anti-vaccination movement (i.e., those who reject vaccination completely and totally), different gradations of radicalism can be discerned. Of the five anti-vaccinationists systematically studied, Nittinger and Förster are the two most radical, outlining a far-reaching conspiracy clearly aimed at the deliberate poisoning of the German people. Just like the introductory example of the Viennese mayor Karl Lueger, Förster in particular can also be clearly located within the *völkisch* ideology; Nittinger also draws heavily in his argumentation on the idea of an entire national body that would be destroyed by vaccination. Given these strong parallels, it is probably no coincidence that both of them used antisemitic narratives and assumed that the Jews are the driving force behind the imagined conspiracy. This overlap of radical anti-vaccination with *völkisch* ideology and antisemitic narratives is a unique feature that distinguishes the German-speaking anti-vaccination movement from movements in other countries. The example of C.G.G. Nittinger also shows that these aspects were part of the rejection of vaccinations from the very beginning – even before the movement became institutionalised after 1874. Nittinger is generally a pioneer in his arguments, as his work predates the actual beginning of *völkisch* ideology.

The less-radical proponents, such as Oidtmann, Molenaar, and Wegener, also resorted to conspiracy theories, but they merely alleged a broad conspiracy to cover up the damage and risks of vaccination. All of the vaccination opponents studied have in common the attempt to give their work at least the appearance of scientificity: they quote each other, let so-called experts express themselves, refer to alleged or actual scientific results, and, above all, fall back on the validity of statistics. Through this, and above all through the recourse to alternative medical concepts and positions from the *Lebensreform* movement, factuality was to be established and a system of knowledge created as an alternative concept to orthodox medicine. This happened in an era in which precisely this scientific medicine was able to establish itself as the hegemonic explanatory model for health, illness, and healing in the first place. Thus, it is a struggle for hegemony and interpretive sovereignty but also for attention. The Imperial Vaccination Act of 1874 was merely a catalyst that contributed to the growth of the movement. The five actors studied are representative in that they can be clearly identified as opinion leaders within the movement and also reflect well the milieu that made up the movement: the academically educated middle class, some members of which even practiced medicine themselves.

Future work could investigate the continuities of this movement, especially after 1945. The German-speaking region in particular was characterised by a particularly high level of rejection of corona vaccination within the EU during the COVID-19 pandemic and strong criticism of measures to contain the pandemic. Studies have shown that the protest movement was recruited particularly strongly from similar milieus as the anti-vaccination movement against smallpox vaccination about 150 years earlier.^90^ This suggests historical roots that run from the nineteenth through the twentieth and into the twenty-first century.

## Footnotes

1 WStLA (Wiener Stadt und Landesarchiv) 1.6.1.B6/2. Ex. – Sitzungen | 7.10.1848-8.5.1919.

2 JAMA. XLIX(13) (1907):1127–9, 1127.

3 See Malte Thießen , Immunisierte Gesellschaft: Impfen in Deutschland im 19. und 20. Jahrhundert (Göttingen: Vandenhoeck & Ruprecht, 2017); Eberhard Wolff , ‘Medizinkritik der Impfgegner im Spannungsfeld Zwischen Lebenswelt-Und Wissenschaftsorientierung’, Medizin, Gesellschaft, und Geschichte, 9 (1996), 79–108 11636509.

4 On Karl Lueger see John W. Boyer , Karl Lueger (1844–1910): Christlichsoziale Politik als Beruf. Vol. 93 (Vienna: Böhlau Verlag Wien, 2010).

5 On the German anti-vaccination movement see: Bernhard J. Hess, ‘Seuchengesetzgebung in den Deutschen Staaten und im Kaiserreich vom Ausgehenden 18. Jahrhundert bis zum Reichsseuchengesetz 1900’ (PhD diss.: University of Heidelberg, 2009); Andreas-Holger Maehle, ‘Präventivmedizin Als Wissenschaftliches und Gesellschaftliches Problem: Der Streit über das Reichsimpfgesetz von 1874’, *Medizin, Gesellschaft und Geschichte* 9 (1990), 127–48; Philipp T. Mayr, ‘Die Impfgegnerschaft in Hessen: Motivationen und Netzwerk (1874–1914)’ (PhD diss.: Philipps-Universität Marburg, 2018); Wolff, *op. cit*. (note 3), 79–108; Eberhard Wolff , Einschneidende Massnahmen: Pockenschutzimpfung und Traditionale Gesellschaft im Württemberg des Frühen 19. Jahrhunderts (Stuttgart: Franz Steiner Verlag, 1998).

6 Christine Binzel and Andreas Link, *The deep roots of vaccine hesitancy in Germany: The 19th-century naturopathic movement*. No. 18109. CEPR Discussion Papers (2023).

7 On the contemporary debates on this vaccination-act see: Carl Jacobi and August Guttstadt , Das Reichs-Impf-Gesetz vom 8. April 1874: Nebst Ausführungs-Bestimmungen des Bundesrathes und der Einzelstaaten (Berlin: Kortkampf, 1875).

8

Malte Thießen , ‘Security, Society, and the State’, Historical Social Research/Historische Sozialforschung, 46, 4 (2021), 211–315, 216–8.

9

Heinz Flamm and Christian Vutuc , ‘Geschichte der Pocken-Bekämpfung in Österreich’, Wiener klinische Wochenschrift, 122, 9 (2010), 265–7510.1007/s00508-010-1379-020559885.

10

Achim Landwehr , Historische Diskursanalyse, Vol. 4 (Frankfurt: Campus Verlag, 2008).

11 Wolff, *op. cit*. (note 3), 83–4.

12 On how people cited religious reasons for their own vaccination decisions, see: Elke Hammer-Luza, ‘“Lässt nicht impfen”. Widerstände gegen die Vakzination in der Steiermark in der ersten Hälfte des 19. Jahrhunderts’, *Virus* (Wien) 1 (2022), 101–30. https://doi.org/10.1553/virus20s101.

13 On C.G.G. Nittinger also see: Alessandra Parodi and Mariano Martini , ‘History of Vaccine and Immunization: Vaccine-Hesitancy Discussion in Germany in XIX Century’, Vaccine, 41, 12 (2023), 1989–9310.1016/j.vaccine.2023.02.02936803870.

14

Frank M. Snowden , Epidemics and Society (New Haven: Yale University Press, 2019) 83–95 ; Allan J. Stewart and Paul M. Devlin , ‘The History of the Smallpox Vaccine’, Journal of Infection, 52, 5 (2006), 329–3410.1016/j.jinf.2005.07.02116176833; A.M. Geddes , ‘The History of Smallpox’, Clinics in Dermatology, 24, 3 (2006), 152–710.1016/j.clindermatol.2005.11.00916714195.

15

Stefan Riedel , ‘Edward Jenner and the History of Smallpox and Vaccination’, Baylor University Medical Center Proceedings, 18, 1 (2005), 21–5. doi:10.1080/08998280.2005.11928028 PMC120069616200144.

16 N. Barquet and P. Domingo, ‘Smallpox: The Triumph Over the Most Terrible of the Ministers of Death’, *Annals of Internal Medicine*, 127, 8 (Part 1) (1997), 635–42.

17 Riedel, *op. cit*. (note 15).

18

Andrew Boylston , ‘The Origins of Inoculation’, Journal of the Royal Society of Medicine, 105, 7 (2012), 309–1310.1258/jrsm.2012.12k044PMC340739922843649.

19

Allan J. Stewart and Paul M. Devlin , ‘The History of the Smallpox Vaccine’, Journal of Infection, 52, 5 (2006), 329–3410.1016/j.jinf.2005.07.02116176833.

20

Janice Parrino and Brian S. Graham , ‘Smallpox Vaccines: Past, Present, and Future’, Journal of Allergy and Clinical Immunology, 118, 6 (2006), 1320–6 10.1016/j.jaci.2006.09.037PMC953382117157663.

21

Edward Jenner , An Inquiry Into the Causes and Effects of the Variolae Vaccinae, or Cow-Pox (London: Vaccin Against Smallpox, 1798).

22 Flamm and Vutuc, *op. cit*. (note 9); Axel C. Hüntelmann , ‘Smallpox Vaccination in the German Empire: Vaccination Between Biopolitics and Moral Economy’, Asclepio. Revista de Historia de la Medicina y de la Ciencia, 72, 1 (2020), 292 .

23 Hüntelmann, *Ibid*.

24 Bernhard J. Hess, *Seuchengesetzgebung in den Deutschen Staaten und im Kaiserreich vom Ausgehenden 18. Jahrhundert bis zum Reichsseuchengesetz 1900* (PhD diss.: University of Heidelberg, 2009).

25

Malte Thießen , ‘Vom Immunisierten Volkskörper Zum Präventiven Selbst: Impfen Als Biopolitik und Soziale Praxis vom Kaiserreich zur Bundesrepublik’, Historische Zeitschrift, 61, 1 (2013), 35–64 ; Thießen, *op. cit*. note (3).

26 On the concept of biopolitics see: Michel Foucault , ‘The Birth of Biopolitics’, in Paul Rabinow and James D. Faubion (eds), Ethics, Subjectivity, and Truth (New York: New Press, 1997), 73–9; idem, *The History of Sexuality Vol. 1: The Will to Knowledge* (New York: Penguin, 1998); idem, *Society Must Be Defended: Lectures at the Collège de France* (New York: Picador Press, 2003); Giorgio Agamben , Homo Sacer: Sovereign Power and Bare Life (Stanford: Stanford University Press, 1998); idem, *State of Exception* (Chicago: University of Chicago Press, 2005).

27 Thießen, *op. cit*. note (25), 35.

28

Philipp Sarasin , ‘Ausdünstungen, Viren, Resistenzen: Die Spur der Infektion im Werk Michel Foucaults’, Österreichische Zeitschrift für Geschichtswissenschaften, 16, 3 (2005), 88–108 .

29

Michel Foucault , Security, Territory, Population: Lectures at the Collège De France, 1977–78 (London: Palgrave Macmillan UK, 2009).

30 On this concept see: K. Ballard and M.A. Elston , ‘Medicalisation: A Multi-Dimensional Concept’, Social Theory & Health, 3 (2005), 228–41; Joan Busfield , ‘The Concept of Medicalisation Reassessed’, Sociology of Health & Illness, 39, 5 (2017), 759–7410.1111/1467-9566.1253828052343.

31

Wolfgang U. Eckart and Robert Jütte , Medizingeschichte: Eine Einführung. 2nd ed. (Cologne; Vienna: Böhlau, 2014).

32 Nadja Durbach has analysed this struggle for the English anti-vaccination movement, particularly from a social perspective: Nadja Durbach , Bodily Matters: The Anti-Vaccination Movement in England, 1853–1907 (Durham: Duke University Press, 2005).

33

Ute Frevert , Krankheit als Politisches Problem 1770–1880: Soziale Unterschichten in Preußen Zwischen Medizinischer Polizei und Staatlicher Sozialversicherung (Göttingen: Vandenhoeck & Ruprecht, 1984).

34

Francisca Loetz , ‘“Medikalisierung”, in Frankreich, Großbritannien und Deutschland, 1750–1850. Ansätze, Ergebnisse und Perspektiven der Forschung’, in Wolfgang E. Eckart and Robert Jütte (eds.), Das europäische Gesundheitssystem. Gemeinsamkeiten und Unterschiede in historischer Perspektive (Stuttgart: Franz Steiner Verlag, 1994), 123–61.

35 Philipp Sarasin, ‘Understanding the Coronavirus Pandemic with Foucault?’, *Foucaultblog* (2020). https://www.fsw.uzh.ch/foucaultblog/essays/254/understanding-corona-with-foucault.

36

Carl Gottlob Gustav Nittinger , Ueber die 50jährige Impfvergiftung des Württembergischen Volkes, Vol. 1 (Stuttgart: Hallberger, 1850).

37
*Ibid*., 75–8.

38 Wolff, *op. cit*. (note 3), 82.

39

Heinrich Oidtmann , Dr. H. Oidtmann als Impfgegner vor dem Polizeigericht: Weshalb Ich Meine Kinder Nicht Habe Impfen Lassen; Eine Verteidigungsschrift (Stuttgart: Bitter, 1877); Paul Förster , Pocken und Schutzimpfung: Im Namen des Deutschen Bundes der Impfgegner Herausgegeben von Paul Förster (Berlin: Vrlg. d. Deutschen Bundes d. Impfgegner, 1900); Heinrich Molenaar , Anti-Vaccinator: Illustriertes Jahrbuch des Internationalen Impfgegner-Bundes 1911 (Vienna: Winkler, 1911).

40 Förster, *ibid*., 173.

41
*Ibid*., 242.

42 Paul Förster, *Deutsche Bildung, Deutscher Glaube, Deutsche Erziehung: Eine Streitschrift* (Berlin: Wunderlich, 1906), 133.

43 Wolff, *op. cit*. (note 3); Hammer-Luza, *op. cit*. (note 12).

44 Oidtmann, *op. cit*. (note 39).

45

Hugo Wegener , Impf-Friedhof: Was das Volk, die Sachverständigen und die Regierung vom ‘Segen der Impfung’ Wissen (Berlin: L. Wegener, 1912).

46 Thießen, *op. cit*. (note 25), 123–30.

47

Robert M. Wolfe and Lisa K. Sharp , ‘Anti-Vaccinationists Past and Present’, The BMJ, 325, 7361 (2002), 430–210.1136/bmj.325.7361.430PMC112394412193361.

48 Molenaar, *op. cit*. (note 39); Hugo Wegener , Der ‘Segen’ der Impfung: Wenig von Vielem. Zusammengestellt und Hrsg. von Hugo Wegener (Berlin: Luise Wegener, 1911); Hugo Wegener , Unerhört!!: Verteidigung und Angriff eines Staatsbürgers. Gegen Kirchner! (Berlin: Wegener, 1911); Hugo Wegener , Impf-Friedhof: Was das Volk, die Sachverständigen und die Regierung vom ‘Segen der Impfung’ Wissen (Berlin: L. Wegener, 1912).

49

Christiabe Arndt , ‘Fearful Narratives: Evidence Production in the Visual Rhetoric of the Historic Anti-Vaccine Movement in the German States’, in S. Ehlers and S. Esselborn (eds.), Evidence in Action Between Science and Society (Abingdon: Routledge, 2022), 203–26.

50

Jens Oliver Krüger und Christine Freytag , ‘Lassen wir Emil als Kind impfen?: Einblicke in den pädagogischen Impfdiskurs des 18. Jahrhunderts’, Zeitschrift für Pädagogik, Nr. 5 (2024), 632–49. 10.3262/ZP0000021 .

51 On this staging of children as a site of symbolic politics, albeit in relation to queerness, see: Lee Edelman , No Future : Queer Theory and the Death Drive (Durham, London: Duke University Press, 2005).

52 Snowden, *op. cit*. (note 14).

53

Martin Dinges (ed.), Medizinkritische Bewegungen im Deutschen Reich: (ca. 1870 – ca. 1933) (Stuttgart: Steiner, 1996).

54

Robert Jütte , Geschichte der Alternativen Medizin: Von der Volksmedizin zu den Unkonventionellen Therapien von Heute (Munich: Beck, 1996), 17–42 .

55

Alex C. Hüntelmann , Hygiene im Namen des Staates. Das Reichsgesundheitsamt 1876–1933 (Göttingen: Wallenstein, 2008).

56

Paul Kübler , Geschichte der Pocken und der Impfung (Berlin: Hirschwald, 1901), 336–336 .

57

Max Kirchner , Schutzpockenimpfung und Impfgesetz: Unter Benutzung Amtlicher Quellen (Berlin: Richard Schoetz, 1911), 100 .

58

Theodore M. Porter , The Rise of Statistical Thinking (Princeton: Princeton University Press, 2020).

59

Theodore M. Porter , Trust in Numbers: The Pursuit of Objectivity in Science and Public Life (Princeton: Princeton University Press, 1995), 147 10.1177/03063129902900400711623934.

60

Axel C. Hüntelmann , ‘Smallpox Vaccination in the German Empire: Vaccination Between Biopolitics and Moral Economy’, Asclepio. Revista de Historia de la Medicina y de la Ciencia, 72, 1 (2020), 292 .

61

Carl Gottlob Gustav Nittinger , Ueber die 50jährige Impfvergiftung des Württembergischen Volkes, Vol. 1 (Stuttgart: Hallberger, 1850); Carl Gottlob Gustav Nittinger , Das Falsche Dogma von der Impfung und Seine Rückwirkung auf Wissenschaft und Staat (Leipzig: Franz, 1857); Carl Gottlob Gustav Nittinger , Das Schwarze Buch vom Impfen: Zeugnisse und Thatsachen, die Impfzeit und die Protestanten gegen Jenner’s Gift und Zauber (Leipzig: Brauns, 1859).

62

Sven Ove Hansson , ‘Science Denial as a Form of Pseudoscience’, Studies in History and Philosophy of Science Part A, 63 (2017), 39–47 10.1016/j.shpsa.2017.05.00228629651.

63 Heinrich Oidtmann, *Der Impfgegner* (1886).

64 Maehle, *op. cit*. (note 5).

65 Thießen, *op. cit*. (note 3), 39–41.

66

Paul Förster , Pocken und Schutzimpfung: Im Namen des Deutschen Bundes der Impfgegner Herausgegeben von Paul Förster (Berlin: Vrlg. d. Deutschen Bundes d. Impfgegner, 1900), 10 .

67

Wolfgang U. Eckart and Robert Jütte , Medizingeschichte: Eine Einführung. 2nd ed. (Cologne; Vienna: Böhlau, 2014), 374–5.

68 On the Lebensreform-movement see: Corinna Treitel , Eating Nature in Modern Germany : Food, Agriculture, and Environment, c.1870 to 2000 (Cambridge : Cambridge University Press, 2017); Eva Locher , ‘Verzicht für Gesundheit und Umwelt: Zur Resonanz der Lebensreform bis heute’, Indes, 9, 4 (2021), 82–90. 10.13109/inde.2020.9.4.82 .

69

Wolfgang U. Eckart , Geschichte, Theorie und Ethik der Medizin (Berlin: Springer-Verlag, 2013), 224 .

70

F.E. Bilz , Das Neue Naturheilverfahren: Lehr- und Nachschlagebuch der Naturgemäßen Heilweise und Gesundheitspflege (Leipzig: Verlag von F. E. Bilz, 1898), 596–9.

71 Förster, *op. cit*. (note 39); Molenaar, op. cit. (note 38); Nittinger, *op. cit*. (note 36).

72 On the Lebensreform-movement see: Eva Barlösius , Naturgemäße Lebensführung: Zur Geschichte der Lebensreform um die Jahrhundertwende (Frankfurt: Campus, 1997); Claudia Huerkamp , ‘Medizinische Lebensreform im Späten 19. Jahrhundert. Die Naturheilbewegung in Deutschland Als Protest Gegen die Naturwissenschaftliche Universitätsmedizin’, VSWG: Vierteljahrschrift für Sozial-Und Wirtschaftsgeschichte, 73, 2 (1986), 158–8211636197; John A. Williams , Turning to Nature in Germany: Hiking, Nudism, and Conservation, 1900–1940 (Stanford: Stanford University Press, 2007).

73 Förster, *op. cit*. (note 39), 3–16.

74

Heinrich Oidtmann , Geschichte der Pocken: Ein Culturkampf der Medicin, Vol. 3 (Frankfurt am Main: Foesser, 1882).

75 On this conflict see: Snowden, *op. cit*. (note 14); Hüntelmann, op. cit. (note 55); Silvia Berger , Bakterien in Krieg und Frieden (Göttingern: Wallenstein, 2009).

76 Nittinger, *op. cit*. (note 36), 58–60.

77

Julia Nebe , Enno Schwanke , and Dominik Groß , ‘The Influence of Epidemics on the Concept of the Bogeyman: Images, Ideological Origins, and Interdependencies of the Anti-Vaccination Movement; The Example of the Political Agitator Paul Arthur Förster (1844–1925)’, Historical Social Research (Köln) 33 (2021), 100–27.

78 Nittinger, *op. cit*. (note 36), 37.

79

Eugen Dühring , Die Judenfrage Als Racen-, Sitten- Und Culturfrage: Mit Einer Weltgeschichtlichen Antwort (Leipzig: Reuther, 1881), 37 .

80

Juliane Wetzel , ‘Bindekitt für Verdrossene und Verweigerer’, in Wolfgang Benz (ed.), Querdenken: Protestbewegung Zwischen Demokratieverachtung, Hass und Aufruhr (Berlin: Metropol, 2021), 55–75 .

81 Nebe et al., *op. cit*. (note 77); Martin Tschiggerl , ‘6. The Other as Conspirator: Historical Roots of COVID-19 Conspiracy Theories’, in H. Steinhoff (ed.), Epidemics and Othering: The Biopolitics of COVID-19 in Historical and Cultural Perspectives (Bielefeld: Transcript, 2024), 119–38.

82

Nico Voigtländer and Hans-Joachim Voth , ‘Persecution Perpetuated: The Medieval Origins of Antisemitic Violence in Nazi Germany’, The Quarterly Journal of Economics 127, 3 (2012), 1339–92.

83 Thießen, *op. cit*. (note 25), 144–5.

84 On the ‘Neue Deutsche Heilkunde’ see Detlef Bothe, *Neue Deutsche Heilkunde, 1933–1945: Dargestellt Anhand der Zeitschrift ‘Hippokrates’ Und der Entwicklung der Volksheilkundlichen Laienbewegung*, No. 62. (Göttingen: Matthiessen Verlag, 1991); Edzard Ernst, ‘“Neue Deutsche Heilkunde”: Complementary/Alternative Medicine in the Third Reich’, *Complementary Therapies in Medicine*, 9, 1 (2001), 49–51.

85 On conspiracy theories from a historical perspective, especially in the 19th century, see: Andrew McKenzie-McHarg, ‘Conspiracy Theory: The Nineteenth-Century Prehistory of a Twentieth-Century Concept’, in J.E. Uscinski (ed.), *Conspiracy Theories and the People Who Believe Them* (Oxford: Oxford University Press, 2019), 62–81; Helmut Reinalter, ‘Verschwörungstheorien im 19. Jahrhundert’, in Helmut Reinalter (Hg.), *Freimaurer und Geheimbünde im 19. und 20. Jahrhundert in Mitteleuropa* (Innsbruck: Studienverlag, 2016), 23–9; Thomas Milan Konda, ‘The Invention of Conspiracy Theory: The French Revolution and the Bavarian Illuminati’, in *Conspiracies of Conspiracies* (Chicago: University of Chicago Press, 2019), 13–27; Claus Oberhauser, Michael Butter, and Peter Knight, ‘Freemasons, Illuminati and Jews: Conspiracy Theories and the French Revolution’, in *Routledge Handbook of Conspiracy Theories*, 1st ed., 1 (Abingdon: Routledge, 2020), 555–68.

86

Michael Barkun , A Culture of Conspiracy: Apocalyptic Visions in Contemporary America, Vol. 15 (Berkeley: University of California Press, 2013).

87

Gilbert T. Cubitt , ‘Conspiracy Myths and Conspiracy Theories’, Journal of the Anthropological Society of Oxford, 20, 1 (1989), 12–26 .

88 Heinrich Oidtmann in Heinrich Molenaar, *Verzeichnis 600 angesehener Impfgegner aus vielen Kulturländern aus dem Adreßbuch circa 1200 tätiger Impfgegner von Dr. med. H. Oidtmann, Linnich (1882) ausgewählt* (Bayreuth: Verlag des Bunds Deutscher Impfgegner, 1914), 28–9.

89 Theodor Hahn, *Die schlimmsten Jesuiten des deutschen Reiches und des deutschen Reichstages: eine öffentliche Denunciation an Sr. Durchlaucht den Fürsten Reichskanzler v. Bismarck* (Sankt Gallen: Altwegg-Weber zur Treuburg, 1875).

90 Nadine Frei and Oliver Nachtwey, *Quellen des ‘Querdenkertums’: eine politische Soziologie der Corona-Proteste in Baden-Württemberg* (Universität Basel: Fachbereich Soziologie, 2021).

## Long descriptions

### Figure 1. Long description

A detailed lithograph framed by two classical pillars. The left pillar is topped with an urn labeled Syphilisation and lists German cities including Wien, München, and Berlin. The right pillar is topped with an urn labeled Vaccination and lists cities including Giessen, Grätz, and Königsberg.

At the top center, a title reads Der Sündenfall des X I X. Jahrhunderts, oder das vaccinatorische Hexenwerk. Below this, a large neoclassical building labeled Academie Jennerismus features a skeleton driving a chariot pulled by a cow. A figure in a crown and robes leads the procession. To the right, a chaotic crowd of people interacts near a tall tower labeled with various social ills like Mörder and Räuber.

The foreground contains a dense row of human heads representing different social classes and professions. To the far left, a satyr-like figure sits near a block of text. To the far right, a winged angel kneels.

The base of the image contains a large block of text listing academic disciplines such as Logik, Psychologie, and Anatomie, followed by the phrase Triumph der Vaccine über die Universitäten. The bottom footer includes statistical data regarding mortality rates in Deutschland and Würtemberg for the years 1754 and 1834, and a publication credit for Verlag von R. Jenni, Bern.

Navigate back to Figure 1..

